# The Neuroexposome: Adding Environmental Dimensions to Dementia Studies in Africa

**DOI:** 10.1002/alz70860_101620

**Published:** 2025-12-23

**Authors:** Mohamed Salama

**Affiliations:** ^1^ The American University in Cairo, Cairo, Egypt; Institute of Global Health and Human Ecology at the American University in Cairo, Cairo, Cairo, Egypt

## Abstract

**Background:**

The determinants of healthy/unhealthy aging are not fully known, but it likely results from a complex interaction between genetic and environmental factors. Possible environmental contributors include air and water pollution and chemicals found in pesticides. One challenge in understanding these factors is that they are lifelong exposure, meaning that through the aging journey, people get exposed to many types of environmental factors; it is, therefore, difficult to know which factors – or combinations of factors – contributed to the aging‐related diseases or health status. Fortunately, new scientific methods allow us to identify many of the environmental risks that people have been exposed to throughout their lives. These new methods can help us figure out what causes diseases and what can lead to healthy aging.

**Methods:**

To embrace the complexity of aging process, a more comprehensive and complex model of the disease is warranted. The “exposome” is defined as the measure of all the exposures of an individual across the lifetime, and how those exposures relate to health. The main advantage of the exposome approach over traditional one‐exposure‐one outcome studies is that it provides an innovative and holistic conceptual framework for the investigation of multiple environmental hazards and their combined effects. As such, an exposome‐wide study could improve our understanding of different environmental factors contributing to aging and their interaction with “internal body responses”, including genetic and metabolomic manifestations.

**Results:**

To be presented

**Conclusion:**

Here we propose an exposome study of aging in an Egyptian cohort, thus also addressing the second limitation noted above.

